# Molecular relationships between Australian annual wild rice, *Oryza meridionalis*, and two related perennial forms

**DOI:** 10.1186/1939-8433-6-26

**Published:** 2013-10-28

**Authors:** Masahiro Sotowa, Kenta Ootsuka, Yuu Kobayashi, Yin Hao, Katsunori Tanaka, Katsuyuki Ichitani, Jonathan M Flowers, Michael D Purugganan, Ikuo Nakamura, Yo-Ichiro Sato, Tadashi Sato, Darren Crayn, Bryan Simon, Daniel LE Waters, Robert J Henry, Ryuji Ishikawa

**Affiliations:** 1 Faculty of Agriculture and Life Science, Hirosaki University, Hirosaki, Aomori 036-8561, Japan; 2 Science of Cryobiosystem, The United Graduate School of Agriculture Sciences, Iwate University, Morioka, Iwate 020-8550, Japan; 3 Faculty of Humanities, Hirosaki University, Hirosaki, Aomori 036-8561, Japan; 4 Faculty of Agriculture, Kagoshima University, Korimoto, Kagoshima 890-0065, Japan; 5 Department of Biology and Center for Genomics and Systems Biology, New York University, New York, NY 10003, USA; 6 Graduate School of Horticulture, Chiba University, Matsudo 648, Matsudo, Chiba 0271-8510, Japan; 7 Research Institute for Humanity and Nature, Kyoto 603-8047, Japan; 8 Graduate School of Life Science, Tohoku University, Sendai, Miyagi 980-8577, Japan; 9 Australian Tropical Herbarium, James Cook University, Cairns, Queensland 6811, Australia; 10 Queensland Herbarium, Brisbane Botanic Gardens Mt Coot-tha, Brisbane, Queensland 4066, Australia; 11 Southern Cross Plant Science, Southern Cross University, Lismore, NSW 2480, Australia; 12 Queensland Alliance for Agriculture and Food Innovation, University of Queensland, Brisbane, Queensland 4072, Australia

**Keywords:** Genetic divergence, Australia, Perennial, Annual, *Oryza rufipogon*, *Oryza meridionalis*

## Abstract

**Background:**

The perennial, *Oryza rufipogon* distributed from Asia to Australia and the annual *O. meridionalis* indigenous to Australia are AA genome species in the *Oryza*. However, recent research has demonstrated that the Australian AA genome perennial populations have maternal genomes more closely related to those of *O. meridionalis* than to those found in Asian populations of *O. rufipogon* suggesting that the Australian perennials may represent a new distinct gene pool for rice.

**Results:**

Analysis of an *Oryza* core collection covering AA genome species from Asia to Oceania revealed that some Oceania perennials had organellar genomes closely related to that of *O meridionalis* (meridionalis-type). *O. rufipogon* accessions from New Guinea carried either the meridionalis-type or rufirpogon-type (like *O. rufipogon*) organellar genomes. Australian perennials carried only the meridionalis-type organellar genomes when accompanied by the rufipogon-type nuclear genome. New accessions were collected to better characterize the Australian perennials, and their life histories (annual or perennial) were confirmed by field observations. All of the material collected carried only meridionalis-type organellar genomes. However, there were two distinct perennial groups. One of them carried an rufipogon-type nuclear genome similar to the Australian *O. rufipogon* in the core collection and the other carried an meridionalis-type nuclear genome not represented in the existing collection. Morphologically the rufipogon-type shared similarity with Asian *O. rufipogon*. The meridionalis-type showed some similarities to *O. meridionalis* such as the short anthers usually characteristic of annual populations. However, the meridionalis-type perennial was readily distinguished from *O. meridionalis* by the presence of a larger lemma and higher number of spikelets.

**Conclusion:**

Analysis of current accessions clearly indicated that there are two distinct types of Australian perennials. Both of them differed genetically from Asian *O. rufipogon*. One lineage is closely related to *O. meridionalis* and another to Asian *O. rufipogon*. The first was presumed to have evolved by divergence from *O. meridionalis* becoming differentiated as a perennial species in Australia indicating that it represents a new gene pool. The second, apparently derived from Asian *O. rufipogon*, possibly arrived in Australia later.

## Background

*Oryza rufipogon* is the recognized wild progenitor of cultivated rice, *Oryza sativa*, and is generally presumed to be distributed from Asia to Australia (Chang 1976, Vaughan 1994). Both annual and perennial forms of wild rice are found in Australia. The perennial form has been regarded as *O. rufipogon*, while the annual form is *O. meridionalis*. The classification of *Oryza* species has been reviewed by Oka (1988). Two species, *O. sativa* and *O. glaberrima*, are known to be domesticated forms. These cultigens have been domesticated from AA genome species within the genus *Oryza*. *O. sativa* was domesticated originally in Asia and thereafter spread widely as a major crop. *O. glaberrima* was domesticated in western tropical Africa with restricted usage. The evolution of the genus *Oryza,* especially the AA genome species, has been a topic of considerable research interest. These species are widely distributed globally and were once regarded as the *O. perennis* complex consisting of geographical races – Asian, African, American, and Oceanian – inhabiting different regions (Morishima 1969). These were subsequently renamed and divided into different species: *O. rufipogon, O. barthii, O. longistaminata, O. glumaepatula*, and *O. meridionalis* (Oka 1988). The wild progenitor of *O. glaberrima* is an annual species, *O. barthii*, widespread in west tropical Africa. The related perennial species, *O. longistaminata*, is more widely distributed in Africa. *O. glumaepatula* is a perennial species in South America, known as floating rice. The culms may break at internodes and the floating fragmented stems have the capability to regenerate shoots and roots (Akimoto et al. 1998). *O. meridionalis* is a species endemic to Oceania including New Guinea and Australia, characterized by an annual life history and morphologically by a short anther length (Ng et al. 1981, Vaughan 1994, Lu 1999). *O. rufipogon* is distributed from Asia to Australia and demonstrates a wide range of life history traits from annual to perennial (Oka and Morishima 1967, Vaughan 1994). The annual form is sometimes referred to as a distinct species, *O. nivara*, which is spread over a wide area from the Deccan Peninsula to the Gangetic valley. Other researchers regard this species as an annual form of *O. rufipogon* (Oka 1974, Sharma 2003). This divergence between annual and perennial types within a single species is found only in *O. rufipogon*. Two species in Africa correspond to these life histories. As *O. meridionalis* from Oceania is regarded as an annual species, taxonomists have defined perennials from that region as *O. rufipogon* (Henry et al. 2010).

Divergence of annual-perennial life history traits has been centered on reproductive systems and longevity. Annuals produce more abundant seeds than perennials before the plants die. In contrast, perennials preferentially propagate vegetatively. *O. meridionalis* grows during the rainy season and dies in the dry season after shedding seeds. This life history trait is the same as that of the annual form of *O. rufipogon.* Perennials are also found in Australia in and around relatively large ponds where water is available continuously throughout the seasons. Sometimes annuals and perennials grow together in the rainy season, usually until around late April. Later, only the perennials remain and propagate vegetatively.

In general, perennials are characterized not only by high vegetative reproduction performance but also marked ability for competitive growth, late flowering, and high outcrossing rates (Sano and Morishima 1982, Morishima et al. 1984). Alternative resource allocations found in annuals result in a low ability to regenerate shoots from nodes, high seed productivity, and short anthers (Oka 1988, Oka and Morishima 1967). These trends for differing resource allocations are generally found among different AA genome species and intermediate types have been found in *Oryza rufipogon* (Sano and Morishima 1982).

The distribution of *O. rufipogon* is known to range from Asia to Australia beyond the biogeographical boundary known as the Wallace line (Wallace 1880, Vaughan 1994). In contrast, the distribution of *O. meridionalis* is restricted and endemic to Oceania. The Wallace line separates the edges of the ancient continents, Sunda (the expanded Eurasian continent including Malaysia) and Saful (the united landmasses of New Guinea, Australia, and Tasmania). It is known that fauna and flora have crossed this line for dispersal. Some fauna migrated across the line when sea levels became lower at the last Glacial Maximum. For example, humans migrated across this line 50,000 ~ 70,000 years ago, when presumably they had not yet acquired agriculture (Bellwood 2005). Flora and fauna have migrated between Asia and Australia over an extended period (Augee and Fox 2012).

Gondwanan elements radiated lineages to form endemic species in Australia. However, more recent immigration from Asia was also extensive. In the fauna, two lizard groups (varanids and gekkonnids) and elapid snakes are known as early immigrants to Australia from South-East Asia. Later immigrants in the mid Tertiary include lizards (scincids and agamids), snakes (typhlopids, boids and acrochorbids), and crocodiles of the genus *Crocodylus*, all of which have close links with South-East Asia. Some frogs, pitted-shelled turtles, snakes and herbivorous bats are relatively recent arrivals as Quaternary immigrants. Plants can move from place to place being spread by wind or by animal vectors. Many tropical plants have moved between South-east Asia and northern Australia. Endemic species in Australia constitute only 14% of the flora in the tropical zone of Australia compared with 47% in the temperate zone in Australia (Augee and Fox 2012). Wild rice, *O. rufipogon*, might have expanded its distribution from Asia to Australia spontaneously or been accidentally transported by animals including humans or birds. This may have happened after the progenitor of *O. meridionalis* had reached Australia, as molecular divergence indicates that *O. meridionalis* diverged from *O. rufipogon* 0.4 to 2 million years ago, long before rice had been domesticated and a dispersal of *O. rufipogon* to the Australian continent (Zhu and Ge 2005, Tang et al. 2010).

Genetic comparison of isozymes among Asian, Oceanian, American, and African forms of AA genome species indicates that the American form, *O. glumaepatula*, is most closely related to the Asian form; the African form is the next closest, and the most distant form is the annual Oceanian form, *O. meridionalis* (Second 1985). Thus, it is difficult to obtain fertile progeny by crossing between Asian *O. rufipogon* and the Australian endemic species *O. meridionalis* (Chu et al. 1969). Nucleotide sequences of particular genes suggest that *O. meridionalis* and *O. longistaminata* have the longest divergence from *O. rufipogon* among the AA genome species (Ge et al. 1999, Cheng et al. 2002, Takahashi et al. 2008). If Australian perennials belong to *O. rufipogon*, they may have resulted from a recent introgression from Asia to Australia, or they represent an independent lineage related to the endemic species, *O. meridionalis* because of their distinct ecological habitats. Complete chloroplast genomes including Asian and Australian perennials have recently been reported (Waters et al. 2012). The chloroplast DNA (ctDNA) of the Australian perennial shares a higher similarity with *O. meridionalis*. This was reconfirmed in this study by analysis of the National Institute of Genetics at Mishima, Japan (NIG) core collection (hereafter, core collection), from which representative accessions were selected and established in a National Bio-Resource Project (NBRP) of Japan. The collection has 1,701 wild rice accessions of 20 species of the genus *Oryza*. Representative accessions of each species have been selected as a core collection representing the typical variation in wild rice (Nonomura et al. 2010). The core collection includes 39 *O. rufipogon* throughout Asia to Oceania with 32 of them originated from Asia, five from New Guinea, and two from Australia. They were regarded as perennials based on their passport data. In addition to the *ex situ* collection, we have worked to collect and evaluate wild rice *in situ* as a new collection which could be accessed to confirm environmental conditions and population structure further. By using the core collection and the new accessions, we have now conducted further detailed characterization of the nuclear genomes of Australian perennials and the relationships between chloroplast DNA (ctDNA), mitochondorial DNA (mtDNA), and nuclear DNA to determine the precise distribution of Asian *O. rufipogon* and to evaluate the unique genetic resources endemic to Australia. In this report, we identified two alternative types of perennial accessions at the morphological and molecular levels in Australia. Both of them were highly divergent from Asian *O. rufipogon* at the organellar DNA (including both ctDNA and mtDNA) level and they showed different morphological and nuclear DNA characteristics from each other. One of the perennial types was referred to as *O. rufipogon*, r-type, perennial and is similar to Asian *O. rufipogon* at the nuclear DNA level. Another type was referred to as an *O. meridionalis,* m-type, perennial and is similar to *O. meridionalis*. We characterized their divergent traits and described these new gene pools in a study that should prove useful in understanding rice evolution and to support genetic improvement of modern cultivars.

## Results

### Newly collected accessions

**Figure 1:**
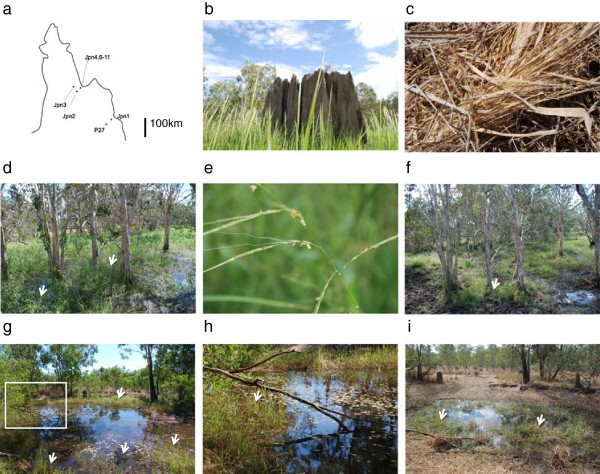
**Typical annual and perennial individuals inhabited in Queensland, Australia. a**. Collection sites in Queensland, Australia. Details were described in Table 1. Approximately a scale bar is 100 km. **b**. Annual population of *O. meridionalis* at the end of rainy season. **c**. Annual rice died and left shedding seeds over ground at Jpn6 site in August, **d**. Perennials inhabited through a year at Jpn1 site. In end of April, there was still water inside the swamp and perennial wild rice plants were grown. Arrow heads showed rice plants. **e**. Close up of perennial rice at Jpn1 site. **f**. There was still muddy at Jpn1 site in August. Around the same tree where wild rice plants were grown at April, perennial wild rice plants survived. Arrow heads showed rice plants. **g**. Perennial wild rice plants found at Jpn2 site at April, 2010. Arrow heads showed rice plants. Part of the population was closed-up in (h). **h**. Arrow heads showing perennial rice. **i**. The population still survived in dry season in August, 2011. Wild rice plants were grown at peripheral zone of the pond. Size of the pond was decreased and alive wild rice plants inhabited at central zone compared to the late rainy season.

Newly collected accessions were collected from north Queensland, Australia at the beginning of the dry season (Figure 1a). These accessions were distinctive from other accessions because they were characterized with the detailed ecological features of each site and with Global Positioning System (GPS) data. Thus, we have referred to them as new accessions to distinguish them from the past accessions. All accessions were named by site name (for example Jpn1). Multiple samples were collected and stocked at the DNA bank at Southern Cross University, New South wales, Australia under approval of EcoAccess published by the Queensland state government. Annual individuals were collected at Jpn6, Jpn7, Jpn8, Jpn9, and P27 (Table 1, Figure 1a, c). In contrast, living individuals were collected at Jpn1, Jpn2, Jpn3, Jpn4, Jpn10, and Jpn11 where perennial populations were found as confirmed by our repetitive observations. The Jpn1 population lives in a swamp (Figure 1d-f), and in the dry season, the swamp was still muddy with some moisture at August (Figure 1d). These conditions are sufficient for perennial vegetation to survive. Panicle morphology clearly showed that they were distinct from *O. meridionalis*. The Jpn2 site was far from the Jpn1 site and located on the Great Dividing Range (Figure 1a). The site was an isolated small pond as shown in Figure 1g-i. In the dry season, there was still plenty of water to support perennial individuals (Figure 1i). The perennial wild rice individuals were found around the pond but not in the central part. This suggested that they have a different strategy to adapt to water condition compared with floating rice. Gradually the water area shrunk in the dry season (Figure 1i). Other details were listed in Table 1. Accessions at the P27 site were classified in this study as *O. meridionalis*, because they behaved as annual wild rice. They did not survive in the greenhouse after flowering.

**Table 1:** Core collection from national bio- resources in National Institute of Genetics and new collection

**Acc. no.**	**No of individuals**	**Species or life history**	**Origin**	**Former description (Katayama with New Guinea collection)**
National bio-resource project, core collection				
W1297	1	*O. meredionalis*	Darwin, Australia	
W1300	1	*O. meredionalis*	Darwin, Australia	
W1625	1	*O. meredionalis*	Darwin, Australia	
W1627	1	*O. meredionalis*	Australia	
W1631	1	*O. meredionalis*	Kununurra area, Australia	
W1635	1	*O. meredionalis*	Darwin, Australia	
W1638	1	*O. meredionalis*	Queensland, Australia	
W2069	1	*O. meredionalis*	Kununurra area, Australia	
W2071	1	*O. meredionalis*	Kununurra area, Australia	
W2077	1	*O. meredionalis*	from Darwin to Normanton, Australia	
W2079	1	*O. meredionalis*	from Darwin to Normanton, Australia	
W2080	1	*O. meredionalis*	from Darwin to Normanton, Australia	
W2081	1	*O. meredionalis*	Matarauka, Australia	
W2100	1	*O. meredionalis*	Queensland, Australia	
W2103	1	*O. meredionalis*	Queensland, Australia	
W2105	1	*O. meredionalis*	Queensland, Australia	
W2112	1	*O. meredionalis*	Queensland, Australia	
W2116	1	*O. meredionalis*	Queensland, Weipa, North Point, Australia	
Asian *O. rufipogon*				
W0106	1	*O. rufipogon*	Phulankara, near Cuttack, Orissa, India	
W0107	1	*O. rufipogon*	Pahala, Orissa, India	
W0108	1	*O. rufipogon*	Cuttack, Orissa, India	
W0120	1	*O. rufipogon*	Cuttack, Orissa, India	
W0137	1	*O. rufipogon*	Kadiam, Andhra, India	
W0180	1	*O. rufipogon*	Ngao, Lampang, Thailand	
W0593	1	*O. rufipogon*	Binjai, Rendah, Malaya	
W0610	1	*O. rufipogon*	Rangoon, Burma	
W0630	1	*O. rufipogon*	Magwe, Burma	
W1294	1	*O. rufipogon*	Musuan, Mindanao Philippines	
W1551	1	*O. rufipogon*	Saraburi, Thailand	
W1666	1	*O. rufipogon*	Siliguri, India	
W1669	1	*O. rufipogon*	Orissa, India	
W1681	1	*O. rufipogon*	Orissa, India	
W1685	1	*O. rufipogon*	Orissa, India	
W1690	1	*O. rufipogon*	Chengrai, Thailand	
W1715	1	*O. rufipogon*	China	
W1807	1	*O. rufipogon*	Sri Lanka	
W1852	1	*O. rufipogon*	Chiang Saen, Thailand	
W1865	1	*O. rufipogon*	Saraburi, Thailand	
W1866	1	*O. rufipogon*	Saraburi, Thailand	
W1921	1	*O. rufipogon*	Saraburi, Thailand	
W1939	1	*O. rufipogon*	Bangkoknoi, Thailand	
W1945	1	*O. rufipogon*	No description	
W1981	1	*O. rufipogon*	Palembang Indonesia	
W2003	1	*O. rufipogon*	from Pajani to Bombay, Indai	
W2014	1	*O. rufipogon*	India	
W2051	1	*O. rufipogon*	Hobiganji, Bangladesh	
W2263	1	*O. rufipogon*	Cambodia	
W2265	1	*O. rufipogon*	Laos	
W2266	1	*O. rufipogon*	Laos	
W2267	1	*O. rufipogon*	Laos	
Australian *O. rufipogon*				
W2078	1	*O. rufipogon*	from Darwin to Normaton, Australia	
W2109	1	*O. rufipogon*	Queensland, Australia	
New Guinean *O. rufipogon*				
W1230	1	*O. rufipogon*		Baad, Koembe, Ducth New Guinea, collected by Katayama (1968) *O. perrenis*
W1235	1	*O. rufipogon*		Baad, Koembe, Ducth New Guinea, collected by Katayama (1968) O. sativa var. spontanea*
W1236	1	*O. rufipogon*		Baad, Koembe, Ducth New Guinea, collected by Katayama (1968) *O. perrenis*
W1238	1	*O. rufipogon*		Baad, Koembe, Ducth New Guinea, collected by Katayama (1968) *O. sativa var. spontanea*
W1239	1	*O. rufipogon*		Baad, Koembe, Ducth New Guinea, collected by Katayama (1968) *O. sativa var. spontanea*
New collection		(Life history)		
Jpn1	3	Perennial	Queensland, Australia (S16.38.085, E145.19.366)	
Jpn2	5	Perennial	Queensland, Australia (S15.26.219, E114.12.397)	
Jpn3	5	Perennial	Queensland, Australia (S15.04.306, E143.43.212)	
Jpn4	3	Perennial	Queensland, Australia (S14.48.481, E143.20.209)	
Jpn6	-	Annual	Queensland, Australia (S14.36.294, E143.55.490)	
Jpn7	-	Annual	Queensland, Australia (S14.42.318, E144.00.130)	
Jpn8	-	Annual	Queensland, Australia (S14.45.458, E144.04.409)	
Jpn9	-	Annual	Queensland, Australia (S14.44.229, E144.04.111)	
Jpn10	4	Perennial	Queensland, Australia (S14.45.244, E144.07.160)	
Jpn11	3	Perennial	Queensland, Australia (S14.50.582, E144.10.055)	
P27	8	*O. meridionalis*	Mareeba (S16.55.02, E145.232.312)	

*O. sativa var. spontanea* which is identical to *O. nivara,* probably a form of *O. rufipogon.*

### Divergent plastid sequences in Australian samples

**Figure 2:**
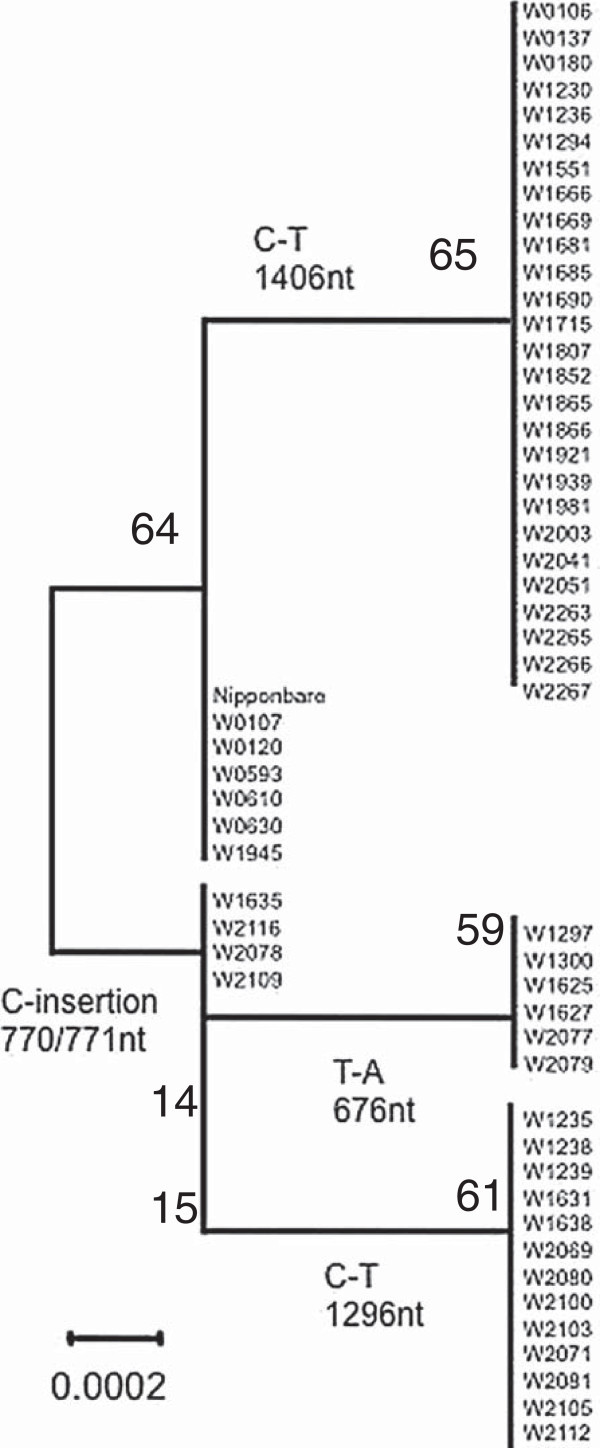
**Polymorphism characterizing the chloroplast genome of Oceania wild rice.** SNP found in the *rpl*16 gene of the ctDNA genome of Asian *O. rufipogon*, Australia *O. rufipogon*, and *O. meridionalis*. T-A substitution (676nt) and C insertion between 770 and 771nt in the 1^st^ intron, C-T substitutions at 1295nt and 1406nt.

Sequences of chloroplast protein coding gene, *rpl16* which includes one intron, were compared within the *Oryza* core collection (Table 2, Additional file 1: Figure S1). SNPs and a C nucleotide insertion were detected as polymorphic sites. The C-nucleotide insertion between 770/771-nt in the gene was found in three out of five New Guinean accessions, two Australian *O. rufipogon*, and 18 *O. meridionalis* (Figure 2).

**Table 2:** Primers used to detect INDEL and SSR

**Primer name**	**Chr**	**Genome position (nt)**	**Forward primer (5’-3’)**	**Reverse primer (5’-3’)**	**Remarks *(O. meridionalisis)***
rp116			AGAAATTCTACCTCTTTCTATAAG	AATTGCCTCGGTAGGATTTTCC	
ORF100-INDEL			GCCGCTTTAGTCCACTCAGCCATC	TCAATGCCTTTTTTCAATGGTCTC	5 bp insert
ctDNA336			ACAGAGGCAAGAAATAACGATTG	TTTATTCTTTCTTTCCAATTTTATG	For genotyping of ctDNA insertions
mtDNA indel			GACTCTGATTCCCCCACTATGAGAGAGCTG	CAGTCCGATGCGTTTGAGCAGTAG’]	4,132 bp deletion
INDEL5	chr10	20772383	AAGTGTGCCTTGCAACCGAG	AAGCAGCAGAACACCTGAAAC	20 bp deletion
INDEL7	chr10	21653822	GTAGCTAGTCGACAGGCAGATG	TACTGGGTATGTAAACCTGCAC	92 bp deletion
INDEL8	chr5	25412945	GATATATTTGTGCTGGCATTCTC	TTCCAGTGAAAATCATATGCAC	33 bp deletion
INDEL9	chr6	28288641	GTGTTCCTAAAACTTATGCATTGTG	CTACCAATTAGCTGTATTAACAAG	60 bp deletion
INDEL10	chr3	32263786	ATTTCAACAGCAGAATGGATTTTC	GAGCAGTTATAGTAACTTGGAGG	155 bp deletion
SSR1	chr3	10541915	GCCACCGAAACTTGTACCGTC	GTAACTTTCTGGTTGTTCCTAAAC	SSR
SSR2	chr6	13061135	CTCCACCGTGAATGTACGTAAG	CGCATCACCTCCTGCAAC	SSR
SSR3	chr10	21872477	CAAAGCTAGCCACTTGCATATG	GTCGTCGACGAACTTGGATAG	SSR
SSR5	chr4	27568462	CTTGTCAACTACTGTGGCAAG	AGAAGATCAACGGTGGTATAG	SSR
SSR7	chr10	15561422	CAAGGATGCTATAAGAGCAAAAAC	TCCTAACACTCCTATTTCATC	SSR
SSR9	chr5	1913	CTGCAAGGATCGCAAACAAAG	TATGGAAATTTGTGCGAGGTG	SSR
SSR15	chr3	34182494	CCTTGGTATTGGTTTGAATIG	TAAAGGATTGCTGGAGAAAGAAG	SSR

This insertion represents a distinct difference between Asian *O. rufipogon* and Oceanian wild rice including some New Guinean *O. rufipogon,* Australian *O. rufipogon,* and *O. meridionalis*. Thus, hereafter the latter type was termed merdionalis- type.

The ORF100-INDEL previous reported as a 5 bp insertion or two 5 bp insertions in *O. meridionalis* was also genotyped. These insertions were always associated with the C insertion (Table 3). Most of the Oceania accessions carried one of the two 5 bp INDELs, while W1631 and W2071 carried two 5 bp insertions (Additional file 1: Figure S1). The remaining *O. meridionalis* accessions carried a single insertion between nucleotide 7998 and nucleotide 7999 in the ctDNA. Thus, it was possible to characterize all of the Australian accessions including Australian *O. rufipogon* and *O. meridionalis* by the C insertion with the ORF100-INDEL when the core-collection was used.

**Table 3:** Variation at ctDNA and mtDNA polymorphism

			**Variation of stretches**								**ORF 100-INDEL**			**mtDNA-INDEL**	
**Site/Collection**	**Species/Accession**	**No. of strains**	**C-absence**					**C-presence**			**-**	**+**		**-**	**+**
			**15A**	**16A**	**17A**	**18A**	**19A**	**11A**	**14A**	**15A**		**+5 bp**	**+10 bp**		
Jpn 1	Australian perennial accesison	3	0	0	0	0	0	0	0	3	0	3	0	3	0
Jpn 2	Australian perennial accesison	5	0	0	0	0	0	0	0	5	0	5	0	5	0
Jpn 3	Australian perennial accesison	5	0	0	0	0	0	0	0	5	0	5	0	5	0
Jpn 4	Australian perennial accesison	3	0	0	0	0	0	0	0	3	0	3	0	3	0
Jpn 10	Australian perennial accesison	3	0	0	0	0	0	0	0	3	0	3	0	3	0
Jpn 11	Australian perennial accesison	3	0	0	0	0	0	0	0	3	0	3	0	3	0
Core collection	*O. meridionalis*	18	0	0	0	0	0	1	14	3	0	16	2	18	0
Core collection	Asian *O. rufipogon*	32	6	8	15	2	1	0	0	0	32	0	0	0	32
Core collection	Australian *O. rufipogon*	2	0	0	0	0	0	0	0	2	0	2	0	2	0
Core collection	New Guinean *O. rufipogon*	5	0	1	1	0	0	0	3	0	2	3	0	3	2

Adenine stretches beside the C insertion in *rp116,* nucleotide in 1st intron of *rp116* and INDEL in mtDNA.

In addition, three SNPs in *rpl16* divided accessions into sub-clades within the species. A C to T (C-T) nucleotide substitution at nucleotide 1406 was found in 25 out of 32 Asian *O. rufipogon* accessions (Figure 2, Additional file 1: Figure S1). Two substitutions, T-A at nucleotide 676 and C-T at nucleotide 1296, divided *O. meridionalis* into three sub-clades. The latter predominated in accessions originating from Queensland. The A stretches beside the C insertion varied from 11 to 19 repeats in total, while *O. meridionalis* showed only three types of A stretches, referred to as 11A, 14A, and 15A (Table 3). In *O. meridionalis*, W2081 carried the 11A type, and three accessions – W1635, W2071, and W2077 – carried the 15A type. Two Asutralian *O. rufipogon* accessions, W2078 and W2109 and three New Guinean *O. rufipogon* accessions, W1235, W1238, and W1239, carried 15A and 14A, respectively, all of which were characterized by the C insertion. Another two New Guinean *O. rufipogon* accessions, W1230 and W1236 carried different repeats, 17A and 16A, respectively, both of which did not carry the C insertion. The meridionalis-type *O. rufipogon* accessions in Oceania carried A-stretches within the variation found in *O. meridionalis*.

**Figure 3:**
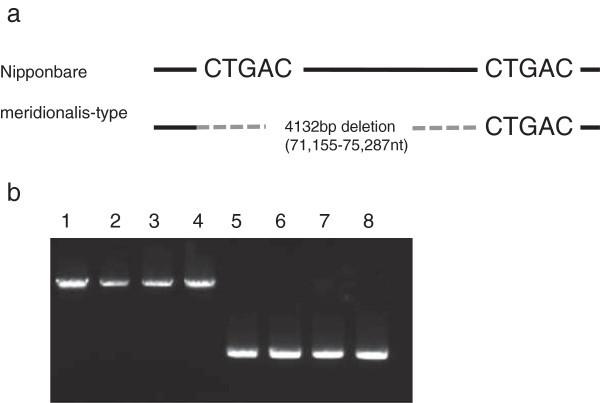
**Deletion in mtDNA genome from nucleotides 71,155 to 75,287 which is found in *O. meridionalis*, three New Guinean *O. rufipogon*, two Australian *O. rufipogon*, and two perennial strains, Jpn1 and Jpn2. a**. meridionalis-type mtDNA showed a deletion via a five base pair tandem duplication located at nucleotides 71,155-71,159 and 75,288-75,292. **b**. PCR amplification detected deletions in meridionalis-type mtDNA. Lane 1 to 8 : Nipponbare, W106, W120, W137, W1299, W1300, Jpn1, and Jpn2.

An INDEL detected in mtDNA was also found to parallel with ctDNA polymorphism (Figure 3, Table 3). Three New Guinean accessions, W1235, W1238, and W1239, carried the meridionalis-type ctDNA polymorphism and had the same mtDNA deletion type as Australian *O. rufipogon* and *O. meridionalis* but the remaining accessions shared similar genomes with Asian *O. rufipogon*.

All perennial accessions in Australia carried the C insertion in *rpl16* along with the insertion in the ORF100-INDEL and the deletion in mtDNA. It was concluded that all Australian perennials carried the meridionalis-type organellar genomes. New Guinean *O. rufipogon* accessions carried either type, rufipogon- or meridionalis-type. The New Guinean accessions were collected in 1960’ (Katayama 1968). Two of them, W1230 and W1236 were defined as *O. perennis* (regarded as current perennial type *O. rufipogon*) and others were as *O. sativa* var. *spontanea* (current annual type *O. rufipogon* or *O. nivara*). As the ecological habit in nature is not certain at present without GPS data, new accessions were further characterized with ecological information to characterize perennial accessions in Australia.

### Genotyping of nuclear DNA

Nuclear INDEL markers were designed to distinguish *O. meridionalis* from Nipponbare (Additional file 2: Figure S2). The 32 Asian *O. rufipogon* accessions all carried insertions at the five loci (Table 4). In contrast, the 18 *O. meridionalis* accessions carried deletions at all loci compared to Nipponbare and other Asian *O. rufipogon*. The genotypes with the deletions were defined as having the meridionalis-type nuclear genome. These INDELs were able to strictly distinguish the rufipogon-type from the meridionalis-type nuclear genomes except for INDEL10. This insertion was homologous with a Stoway DNA transposon and one Australian accession in the core collection showed the deletion type. However, other accessions regarded as *O. rufipogon* had the insertion. These INDELs were used to characterize the Australian perennials.

**Table 4:** **Nuclear DNA INDEL genotypes among newly obtained accessions, *O. rufipogon *** **originated from Asia, Australia, and New Guinea, and *O. merdinoalis***

**Site/Collection**	**Species/Accessions**	**No. of accessions**	**Nuclear DNA INDEL genotypes**				
			**INDEL5**	**INDEL7**	**INDEL8**	**INDEL9**	**INDEL10**
Jpn1	Australian perennial accessions	3	+	+	+	+	+
Jpn2	Australian perennial accessions	5	-	-	-	-	-
Jpn3	Australian perennial accessions	5	-	-	-	-	-
Jpn4	Australian perennial accessions	3	-	-	-	-	-
Jpn10	Australian perennial accessions	3	-	-	-	-	-
Jpn11	Australian perennial accessions	3	-	-	-	-	-
Core collection	*O. meridionalis*	18	-	-	-	-	-
Core collection	Asian *O. rufipogon*	32	+	+	+	+	+
	Australian *O. rufipogon* W2078	2	+	+	+	+	+
Core collection	Australian *O. rufipogon* W2109	2	+	+	+	+	+
Core collection	NG *O. rufipogon*: W1230, W1236, W1238	3	+	+	+	+	+
	NG O. *rufipogon*: W1235, W1239	2	-	-	-	-	-

The new perennial accessions, except for those from the Jpn1 site, showed deletions at the five loci. They were genetically similar to *O. meridionalis* at the nuclear DNA level, even though this species is known to be an annual, and the collected accessions were all perennial. However, three accessions at the Jpn1 site showed genotypes without the deletion at all loci like Asian *O. rufipogon,* although they carried the meridionalis-type organellar genomes. Their nuclear genomes were determined to be rufipogon-type. This suggested that the Jpn1 accessions were genetically similar to *O. rufipogon* at the nuclear DNA level, although they shared the same meridionalis-type organella genome as the other Australian perennials. Thus, it was concluded that there are different perennial types in Australia, r-type and m-type. The former carries rufipogon-type nuclear genome and meridionalis-type organelle genomes. The latter carries all meridionalis-type including both nuclear and organelle genomes. Both r-type and m-type carried meridionalis-type organelle genomes demonstrating their divergence from Asian *O. rufipogon*.

Seven SSR loci were used to compare species diversity and phylogenetic relationships among Asian *O. rufipogon*, *O. meridionalis*, New Guinean *O. rufipogon*, and Australian perennials (Table 5). *Fst* scores for the seven loci ranged from 0.21 to 0.80 (mean ± SD, 0.53 ± 0.09). They did not diverge as much as the nuclear INDEL markers (*Fst* = 0.84 at all loci examined). The total number of alleles per loci ranged from three to ten; the average number of alleles was 5.43. Examination of heterozygosity (*He*) revealed that these loci were moderately polymorphic. SSR2 showed the highest polymorphism, 0.83, and SSR7 showed the lowest, at 0.50.

**Table 5:** **Fst, numbers of alleles (*Na *), and Heterozygosity in SSR markers among three groups, rufi (Asian *O. rufipogon*, n = 32), meri (*O. meridionalis *, n = 18), NG (New Guinean, n = 5), AUS (Australian *O. rufipogon*, n = 5) Jpn1 accessions (n = 3), and Jpn2-11 (accessions excluded Jpn1 site which obviously shared a similarity with *O. rufipogon *** **by nuclear INDELs, n = 20) with variation including all listed in Table**1

**SSR locus**		***Na*** **(n=)**							***He***						
	**Fst**	**Total**	**Asian rufi**	**meri**	**NG**	**Aus**	**Jpnl**	**Jpn2-11**	**Total**	**Asian rufi**	**meri**	**NG**	**Aus**	**Jpn1**	**Jpn2-11**
SSR1	0.75	4	3	1	3	1	1	1	0.57	0.36	0.00	0.64	0.00	0.00	0.00
SSR2	0.21	10	6	5	4	2	1	2	0.83	0.71	0.68	0.70	0.38	0.00	0.32
SSR3	0.80	3	3	1	2	1	2	1	0.51	0.12	0.00	0.48	0.00	0.44	0.00
SSR5	0.54	5	4	1	4	1	1	1	0.60	0.66	0.00	0.72	0.00	0.00	0.00
SSR7	0.26	4	4	2	2	1	1	1	0.50	0.60	0.49	0.32	0.00	0.00	0.00
SSR9	0.50	9	7	1	3	1	3	1	0.66	0.67	0.00	0.64	0.00	0.67	0.00
SSR15	0.69	3	2	2	2		2	1	0.63	0.17	0.28	0.48	0.00	0.44	0.00
No. of accessions		80	32	18	5	2	3	20							
Mean	0.54	5.43	4.14	1.86	2.86	1.14	1.57	1.14	0.62	0.47	0.21	0.57	0.05	0.22	0.05
±SE	0.09	1.09	0.67	0.55	0.34	0.14	0.30	0.14	0.04	0.09	0.11	0.06	0.05	0.11	0.05

Na (n=): No. of alleles and number of accessions including each group.

*O. meridionalis* group had four monomorphic loci, although Asian and New Guinean *O. rufipogon* groups showed polymorphism at all the loci. The number of alleles ranged from one to five in *O. meridionalis*, and from two to seven in Asian *O. rufipogon*. Averaged *He* scores were 0.47 ± 0.25 (mean ± standard error) for Asian *O. rufipogon*, 0.57 ± 0.06 for New Guinean *O. rufipogon*, and 0.21 ± 0.28 for *O. meridionalis*. These basic data showed that the perennial *O. rufipogon* population tends to have higher diversity as does the New Guinean population. However, in the case of *O. rufipogon*, the accessions were collected in different Asian countries (Table 1). Thus, the *He* score would not reflect that in a natural population, but the general trend in a core collection.

**Figure 4:**
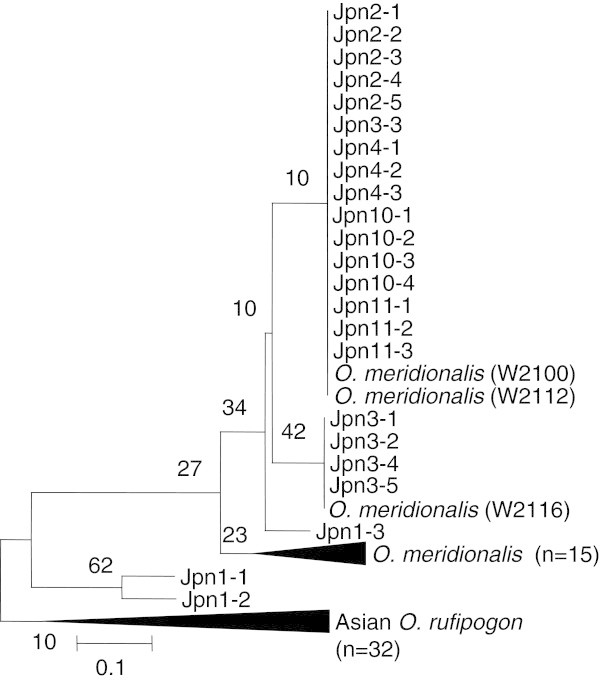
**Phylogenetic tree by NJ method based on genetic distance measured with SSR genotypes.** Compressed nodes shown as triangles were for *O. meridionalis* and Asian *O. rufipogon*. The former node included 15 accessions and another node included 32 accessions. Numbers indicate bootstrap values of (%) with assessment of 1000 replicates. Scale was Nei’s genetic distance (Nei et al. 1983).

Twenty-three Jpn accessions were clearly grouped by their nuclear DNA INDEL genotypes (Table 3). Thus, *Na* and *He* were calculated using two sets of new collections, Jpn1 group and Jpn2-11 group. The Jpn1 group was monomorphic at SSR1, SSR2, SSR5, and SSR.7. The Jpn2-11 group was monomorphic at six loci except for SSR2. A phylogenetic tree based on SSR genotypes was constructed using the neighbor-joining method (Figure 4). Population-based trees showed higher similarity between *O. rufipogon* and Jpn1. Accessions of *O. rufipogon* and Jpn1 were grouped in a single clade except for one accession, Jpn1-3, which was in a clade with most of the *O. meridionalis* accessions. Other Jpn accessions collected from Jpn2, Jpn4, Jpn10, and Jpn11 were in a clad with W2100, W2112, W2116, and all other *O. meridionalis* accessions originating from Queensland.

### Morphological traits

Morphological traits relating to life history such as anther length, seed size and traits involving to seed productivity, were measured for the two perennial groups carrying the different nuclear genomes. The two perennials were compared with the typical annual accessions collected at the P27 site which was close to the Jpn1 site and defined as typical *O. meridionalis* types based on repetitive field observations.

**Figure 5:**
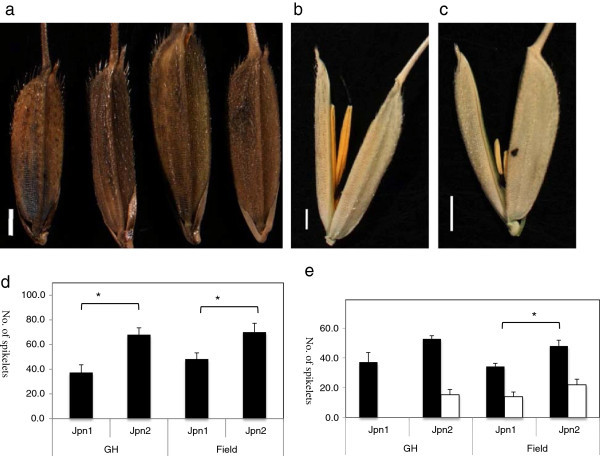
**Phenotypic difference among Australian perennials (Jpn1 and Jpn2 ), and Australian annual (P27, S16.5502, E145.2323, collection in Mareeba, Queensland).** All scale bars are 1 mm. **a**. Spikelets of Jpn1, Jpn2, and P27 (left to right), compared with Asian *O. rufipogon* (W137) as a control. P27 was a typical annual strain belonging to *O. meridionalis*.A: hulls, **b**. Spikelet of Jpn1 carrying relatively longer anther compared to other types of Australian wild rice. **c**. Spikelet of Jpn2 carrying a shorter anther, similar to *O. meridionalis*. **d**. Number of spikelets obtained from Jpn1 and Jpn2 grown under glass house and field conditions. Repetitive number was four panicles. Standard Error bars are shown. Number of spikelets differed significantly between Jpn1 and Jpn2 (P = 0.05) under either condition. **e**. Number of spikelets on the 1^st^ rachis (black bars) and the 2^nd^ rachis (open bars) obtained from Jpn1 and Jpn2 grown under green house and field conditions. Standard error bars are shown. When compared with the number of spikelets on the first rachis, there was a significant difference (P = 0.05) only under field conditions. The number of spiekelts on the second rachis differed significantly in Jpn1 and Jpn2, only under glass house condition (P = 0.05).

Spikelet size varied hugely in Jpn2 compared to the others (Figure 5a). Distinct traits of Jpn2 were the large spikelet size, large number of spikelets per panicle and awn size. The lemma size of Jpn2 was 9.14 ± 0.53 (standard deviation) mm was significantly larger (P < 0.01) than that of others. P27 carried the same organellar and nuclear genomes as the population at Jpn2 but had a relatively short lemma like Jpn1. This trait was regulated genetically, as plants grown under glass house conditions confirmed these differences.

Perennials tend to have longer anthers and lower seed productivity. The former trait is associated with the out-crossing rate and the latter trait may interact as a trade-off with vegetative reproduction. As a species defining characteristic, *O. rufipogon* in Australia has been described as having an anther longer than 3 mm. The population at the Jpn1 site had a significantly longer anthers, 5.02 mm in length under field conditions compared to those of *O. meridionalis* and populations at the Jpn2 site (Figure 5b and c, Table 6). These relative relationships were shown to be genetic by confirmation when grown under glass house conditions.

**Table 6:** Morphological diffferences between the two types of perennial

	**GH**			**Field**				
**Traits**	**Jpn1**	**Jpn2**	**t-test**^**#**^	**Jpn1**	**Jpn2**	**t-test**	**P27**	**t-test against to Jpn1**
Anther length (n = 5)								
Mean (mm)	4.03	1.68	**	5.02	2.05	**	1.76	**
SD	0.22	0.27		0.17	0.10		0.24	
Lemma length (n = 4)								
Mean (mm)	7.18	8.96	**	7.44	9.14	**	7.19	ns
SD	0.36	0.10		0.38	0.53		0.57	
Panicle length (n = 4)								
Mean (cm)	15.15	16.38	ns	23.13	19.68	ns	26.30	ns
SD	3.04	2.17		2.14	1.90		4.80	
No. of spikelets per panicle (n = 4)								
Mean	37.3	68.0	*	48.3	70.0	**	46.3	ns
SD	12.7	11.0		10.0	14.4		3.4	
Awn length (n = 20)								
Mean (cm)	-	-		7.60	10.23	**	6.76	*
SD				0.86	0.55		0.84	

Seeds and panicles of Jpn1, Jpn 2, and P27 were collected in the field. Seeds of Jpn1 and Jpn2 were grown under glass house (GH) condition and harvested to measure traits. Repetitive counts (n) were average and the mean and standard deviation were presented as mean and SD.

#: t-test was used to test differences against Jpn1. Significant levels were indicated as * (P = 0.05) and ** (P = 0.01).

Awn length in Jpn2 was 10.23 ± 0.55 cm but those of Jpn1 and P27 were 7.60 ± 0.86 cm and 6.76 ± 0.84 cm, respectively. We could not measure awns under glass house condition because bagging of panicles so as to not to disperse the seeds from spikelets restricted the development of the awns. The most distinctive trait for P27 was panicle size. P27 individuals carried the longest panicles even though they produced the smallest number of spikelets under field condition.

The number of spikelets per panicle for Jpn2 was 70.0 ± 14.4 under field conditions (Figure 5d). This was significantly more than that of Jpn1 (P < 0.05). This trend may be independent of factors such as the nitrogen in the soil because the difference was reconfirmed with plants grown under glass house condition (Table 6). Individuals at the Jpn1 site had a reduced number of spikelets, when they were grown under glass house condition. This mainly resulted from a reduction of secondary spikelets. It may be not have been warm enough to generate secondary spikelets, as we observed at heading in October in Japan. The number of spikelets was increased on the first rachis both in Jpn1 and Jpn2. Fertilization may be affected only by the number of spikelets on the first rachis.

## Discussion

### Nomenclature of *O. meridionalis*

Generally, annual species, *O. meridionalis*, and perennial species, *O. rufipogon* have been known in Australia as the *Oryza* species belonging to AA genome (Henry et al. 2010, Vaughan 1994, Waters et al. 2012). However, in Australia we have not observed an annual form of *O. rufipogon*, which is sometimes referred to as *O. nivara* (Sharma 2003). Only *O. meridionalis* is found as an annual species. In this report, we clearly identified a novel perennial form very close to *O. meridionalis* and distinct from *O. rufipogon*.

One of the obvious morphological differences between *O. meridionalis* relative to *O. rufipogon* is anther length. It has been noted that *O. meridionalis* should have an anther length of less than 1.5-2.5 mm whereas *O. rufipogon* should have a longer anther at 3–7.4 mm (Waters et al. 2012). Our observations revealed that only the plants at Jpn1 had relatively longer anthers (Table 6). Individuals from the Jpn2 and Jpn3 sites showed anther lengths shorter than those from Jpn1, in August 2010. The short anther was not influenced by environmental factors because glass house data indicated the traits were confirmed repetitively in the case of Jpn1 and Jpn2 accessions. In spite of these characteristics, individuals at the Jpn2 and Jpn3 sites survived throughout the dry season. Their resource allocation allowed nodes to generate shoots and roots in water (Additional file 3: Figure S3). Thus, the criterion of anther length is not applicable to classification of Australian wild rice based upon life history traits. Other traits, lemma size, number of spikelets per panicle, and panicle length were different between P27 accessions (*O. meridionalis*) and Jpn2. Generation of a second rachis in Jpn2 resulted in a higher number of spikelets although they did have a shorter panicle length. Thus, the novel accessions are quite divergent to *O. meridionalis*. It infers that the novel perennial type could be a new species. Cross fertility is now being confirmed.

All *de novo* accessions in Australia were classifiable as *O. meridionalis* based on organellar genomes (Table 3). The sequences at both *rpl16* and INDEL genotype in ctDNA revealed diagnostic plastid types, which could be further supported by whole-genome sequence data for the plastid genome of *O. meridionalis* (Waters et al. 2012). This trend was also seen in mtDNA (Figure 3). Simple structural change was fixed in Australia among not only all accessions but also Australian accessions in the core collection. Asian accessions did not show any changes at the site. Nuclear INDEL genotypes clearly distinguished *O. meridionalis* from Asian *O. rufipogon* and *O. sativa*. Five accessions of *O. rufipogon* originating in New Guinea were quite distinct from Asian accessions. Because they had various combinations of nuclear-plastid/mitochondrial types even they had been collected at a single swamp (Katayama 1968). Although these accessions have been defined as *O. rufipogon*, the primarily species definitions were *O. perennis* and *O. sativa* var. *spontanea* which had been used as annual wild rice. Based on SSR genotypes, three were included in a clade with *O. rufipogon*. One of the three accession, W1238 also carried rufipogon-type nuclear INDELs but meridionalis-type organelle genomes like Jpn1 accessions (Table 3). Two carried meridinoalis-type nuclear and organella genomes. They might belong to *O. meridinoalis*. Additional accessions along with ecological information for New Guinea are needed to evaluate their genetic characteristics in detail. All the new Australian perennial accessions carried meridionalis-type organellar genomes. However, there are alternative types of perennials based on their nuclear genotypes.

### Genetic divergence among species

Genetic similarity in nuclear genomes was estimated by INDEL analysis and SSR genotyping (Tables 4 and 5). Annuals were dead at the time of collection and had already scattered their seeds on the ground (Figure 1c). In contrast, all perennials survived through rainy season (Figure 1d and g) to dry season (Figure 1f and i). Repetitive observations at the same sites confirmed their life history traits. Those individuals surviving in the dry season were regarded as perennials, and morphologically different from *O. meridionalis*. However, all accessions collected from the perennial populations shared the same meridionalis-type organellar genomes.

Our genetic data for the perennial AA genome accessions revealed distinct characteristics that have never been found in Asia when compared with the core collection examined in this study. At the molecular level based on ctDNA, all accessions shared DNA polymorphisms with *O. meridionalis.* On the other hand, the nuclear genotypes clearly divided the accessions into the Jpn1 type and an alternative type. The Jpn1 type was included in a clade with Asian *O. rufipogon*, but the alternative was in a clade with *O. meridionalis* (Figure 5). Phylogenetic trees based on SSR genotypes showed that a Jpn1-3 accession was close to *O. meridionalis* in the clade and also to Jpn2 type perennial accessions. In contrast, five nuclear INDEL genotypes clearly indicated that the Jpn1-3 accession at Jpn1 site was defined as belonging to the Asian *O. rufipogon* group (Table 4). This discrepancy would be due to hyper-variation essential to nuclear SSR repeats. Seven SSR loci were not enough to obtain a phylogenetic tree with high bootstrap values on each node.

### Evolutionary pathway for generation of Australian perennials

These results reveal that there are two types of perennials in Australia, r-type and m-type. Then, when did the two perennial types evolve? The question remains to be resolved.

The following possible models can be envisaged:

1) A second migration model: parallel evolution with a second migration of the r-type perennial.

2) Hybrid species (the r-type perennial) and parallel evolution.

3) Hybrid species followed by divergence.

The second migration model presumes that the r-type perennial might have originated from an ancestor that evolved in Asia, which was also a contributor to the Asian *O. rufipogon* gene pool because our recent crossing experiment has shown that Jpn1 can generate fertile progeny in crosses with Asian wild rice (data in preparation). *O. meridionalis* may have been in Australia long before the r-type. Populations related to the ancestor of the r-type perennial may now be extinct in Asia, or have evolved to become modern *O. rufipogon* because we have not found the Australian r-type in Asian genetic resources when we have screened the core collection and other material. Independently from the r-type perennial, the m-type perennial could have been the result of adaptive divergence from a common ancestor to *O. meridionalis* to occupy a particular niche where annuals could not fit. Our preliminary data on reproductive barriers indicates that Jpn2 does not produce much fertile pollen in hybrids with *O. rufipogon* (< 10%) or *O. meridionalis* (< 20-40%)*.* This pollen fertility produced no seeds in glass house. A reproductive barrier may have developed between the annual and perennial populations. However, because they share an ancestral lineage, they have similar morphological and molecular signatures.

Hybrid species with parallel evolution is the second option. Hybrid species might have been generated by a spontaneous cross between Asian *O. rufipogon* and *O. meridionalis*. This could explain the origin of the r-type perennial, but the m-type perennial might be the result of adaptive convergence, as in the above option. A possible date for the introgression of Asian *O. rufipogon* is 50,000 to 70,000 years ago when an ancient continent, Sunda, was located close to Sahul because of a lowered sea level at that time. The rufipogon-type nuclear genotypes with an meridionalis-type organella genomes might have predominated under the prevailing environmental conditions. Hybrid fertility between Asian *O. rufipogon* and *O. meridionalis* is as low as 10% (Chu et al. 1969). Once hybrid species occur in nature, subsequent backcrosses may be required to create fertile progeny. From the current r-type perennial genome constitution it is presumed that pollination from Asian *O. rufipogon* to *O.meridionalis* occurred. This would have yielded m-type organellar genomes and Asian *O. rufipogon* nuclear genomes in a distinct species. Perennials prefer to outcross and retain their genetic diversity to a greater degree than annuals and annuals prefer to self-pollinate (Oka 1988). Once hybrids were created, this plant may have been a highly competitive perennial (with some heterosis) that continued to interbreed with the *O. rufipogon*-like population in the same unidirectional way. When we screened more individuals in Australia, no individual carrying the rufipogon-type organellar genomes was found. We are unable to explain why there was no perennial with an rufipogon-type organelle genomes because currently there is no evidence that the organelle genomes of *O. meridionalis* confers any advantage over the Asian *O. rufipogon* type. We would also expect heterozygotes or heterogeneous genotypes at some loci in this case. However, we have not yet detected such perennials.

In the last model, both the r-type and m-type perennials shared the same incipient progenitor as a spontaneous hybrid between Asian *O. rufipogon* and *O. meridionalis.* If so, we would expect heterozygous or heterogeneous genotypes at some loci or mixtures of plastid types within populations. However, all of the accessions examined carried a rufipogon-type or a meridionalis-type nuclear genome with an m-type organellar genome. We have not found any Jpn2-type perennials in the Northern Territory. The rufipogon-type genomes spread in northern Australia from Northern Territory to Queensland because previous specimens collected in the Northern Territory such as W2078 and also in Queensland as W2109, belong to the rufipogon-type perennial group. The hybridization would need to have occurred sufficiently long ago for the genome constituents to stabilize, to spread over this wide range, and gain complete fertility. This does not explain why the meridionalis-type organellar genomes became predominant in both lineages and we do not have any evidence for this event.

The current distribution of Australian *O. rufipogon* is quite broad, as is that of *O. meridionalis* (Henry et al. 2010)*.* Therefore the presumed parents must have arrived quite a long time ago in each case. Thus, the recent cultivated rice species, *O. sativa,* cannot be the parent of these species. Nevertheless, *O. sativa* is a domesticated species related to *O. rufipogon*, and exchange of their genes by spontaneous crosses resulted in weedy rice (Oka and Chang 1959). There are a few records indicating that domesticated *O. sativa* was briefly cultivated in these areas of Australia, although cultivation did not persist because of bird attacks (Waters et al. 2012). Only the Jpn1 perennial accessions share similarity of nuclear genotypes with Asian *O. rufipogon* and *O. sativa*. Jpn1 is close to Cairns city in distance, where Asians (especially Chinese and Japanese) immigrated in the late 19th century. However, two hundred years might not have been long enough for wild rice to have stabilized after natural hybridization without any sign of domestication traits. Furthermore, perennial types have also been found in Northern Territory and Queensland to carry the Asian *O. rufipogon*-type nuclear genome by estimation of *pSINE1* insertion (Xu et al. 2005). Our data also showed that it carried the meridionalis-type organellar genomes with the rufipogon-type nuclear genome. Thus, perennials with the rufipogon-type nuclear genome have dispersed in unrestricted areas of northern Australia. They cannot be attributed to recent and regional introduction of particular kinds of Asian cultivated rice. It is therefore unlikely that Jpn1 is the progeny of weedy rice resulting from crossing between Australian wild rice and Asian cultivars. The morphological appearance of the Australian populations suggests that they are not the progeny of cultivated rice. If Australian wild rice had been crossed with domesticated rice, specific characteristics would have been generated as weedy rice such as non-shuttering trait, white grain, high sterility in seed setting, or thick stem (Ishikawa et al. 2002, 2005). Such characteristics have never been found in Jpn1 site. Thus, Jpn1 is not a remnant of cultivars like weedy rice. Birds might act as a vector to bring seeds to northern Australia. In addition, the appearance of Jpn2 is not similar to that of Asian *O. rufipogon* from Asia. We were able to detect highly sterile populations in Weipa, northern Queensland, where annual and perennial individuals were found together. They are possibly progeny of recent hybridization between Jpn1 type and *O. meridionalis* because of their higher sterility based on the field observation.

Due to genetic distance and preliminary results of hybridization tests, the second migration or hybridization models seem to provide a possible explanation. The former model, however, would have more probability because we have not found remnant of hybridization events at the molecular level. Thus, we concluded that our second migration model would be appropriate to explain the nature of divergence of *Oryza* species in Australia. In order to draw a firm conclusion, more data on genome constituents compared to known species, divergence time, and evidence of reproductive relationships are needed. The two alternative perennials, the Jpn1 and Jpn2 groups, are probably best considered as new and distinct species that diverged a long time ago.

### Genetic reservoir

Wild rice belonging to the AA genome is a genetic reservoir for use in improving cultivars so that they can be more resistant to biotic and abiotic stress. *O. meridionalis* has been used to establish chromosomal segment introgression lines (Yoshimura et al. 2010). Such introgressions have been used to introduce useful genes from wild AA genomes, and sometimes generate plants of different ploidy or with different genomes (Khush 1997). For *O. australzensis*, genes conferring insect resistance, such as *Bph10* and *Bph18*, and fungus resistance, such as *Pi40*, have been reported (Ishii et al. 1994, Jena et al. 2006, Jeung et al. 2007). *O. meridionalis* and other species in Australia have not been exploited significantly in rice breeding. Because of the limited collections of this material, it has not been possible to evaluate genetic polymorphism for annual populations of *O. meridionalis* or perennial populations such as the Jpn1 and Jpn2 types in their natural habitat. Sympatric habitats, where annual and perennial populations are growing together, are common in north Queensland. A potential hybrid population has been found in Lakeland. Although they may have low fertility, perennials tend to outcross because of their preference for resource allocation. Such potential hybrids or relatives would be good candidates for improving domesticated species. Bridging breeding can be used to efficiently introduce alien segments of genomes to modern varieties. Australian perennial wild rice populations will soon be evaluated more precisely for their genetic characteristics under *in situ* conditions. Intermediate type would be found because of the probable hybridization event in Weipa. In addition to that, artificial crossing experiment will show us how intermediate type behaves in nature or conditioned environment. Extra efforts to ensure conservation of the native habitat of these species have been suggested in order to facilitate continued research on these valuable resources under *in situ* conditions.

## Conclusion

Analysis of current accessions clearly indicated that there are two distinct types of Australian perennials.Both of them differed genetically from Asian *O. rufipogon*. One lineage, m-type is closely related to *O. meridionalis* and another, r-tpe to Asian *O. rufipogon*. The m-type was presumed to have evolved by divergence from *O. meridionalis* based on molecular nature. It is the first accession which has never been found in the former collection. The m-type shared morphological similarity to some extent but differed in spikelet size. Shorter anther of the m-type and *O. meridionalis* is a key character of annual species tended to self-pollinate. However, its life history observed in nature was not annual. It is becoming differentiated as a perennial species in Australia indicating that it represents a new gene pool. The r-type apparently derived from Asian *O. rufipogon*, possibly arrived in Australia later, because of its nuclear genotypes sharing higher similarity to Asian *O. rufipogon*. Maternal genomes suggested r-type, m-type, and *O. meridinoalisi* shared a single lineage. It will give us a clue to understand rice evolution and expansion into a new continent.

## Methods

### Plant materials

Wild rice was collected in Australia with permission from the Queensland government, under the EcoAccess program. We developed these collections as *de novo* resources, which can be accessed repeatedly from the same site with accurate GPS data, allowing us to reconfirm their life cycles. Successive observations were carried out from 2009 until 2011, and the life history traits of plants at the collection sites were reconfirmed year by year. The field research was supported by overseas scientific research funds (JSPS) and collaborative research with Queensland Herbarium and QAAFI, University of Queensland. Observations of the ecological habitat and life cycle of each population in April 2008, August 2009, and September 2009 were used to determine whether each population was annual or perennial. In total, 23 perennial individuals were collected at six sites (Table 1, Figure 1a). Annuals were observed at the Jpn6, Jpn7, Jpn8, Jpn9, and P27 sites. Jpn6 was a typical annual site, as shown in Figure 1a and 1b. In the dry season, the fields dried out completely and individual plants dispersed their seeds. Jpn1 and Jpn2 were typical perennial sites, and individual plants survived in a swamp (Jpn1) or pond (Jpn2) where constant water supply was available (Figure 1c to f). Due to the objectives of this study, only perennial individuals were studied further. One individual per site was pressed and dried to donate as a herbarium specimen to the Queensland herbarium. DNA from the 23 individuals was extracted from dried leaves and some of the DNA materials were stocked in the DNA bank of Southern Cross University. Jpn1 and Jpn2 accessions as representatives of alternative perennials were characterized for anther length, awn size, spikelet size, number of spikelets, and panicle size. Both of the perennials were replanted in pots using a general nursery procedure for cultivated rice under glass house condition at Kagoshima University with natural day length. These plants were also examined for the above morphological traits. Data was obtained for single individuals. The length of anthers in single spikelets was measured. Anther length and lemma size were measured with a NIKON digital sight.

Thirty-two Asian *O. rufipogon*, two Australian *O. rufipogon*, five New Guinean *O. rufipogon*, and 18 *O. meridionalis* accessions from the NIG core collection (hereafter core collection) established at the National Institute of Genetics, Mishima, Japan, were used as controls (Nonomura et al. 2010). The DNA samples were kindly supplied by the National Institute of Genetics. Phenotypic characteristics and phylogentic relationships among some of these strains have been described by Morishima (1969).

### Identification of plastid type from the rpl16 sequences and INDELs in ctDNA and mtDNA

Sequences of rpl16 were used for comparison of maternal origin. Two pairs of primers (rpl16 primers forward and reverse) shown in Table 2 were used to amplify a whole region of *rpl16* gene. Amplified fragments were purified with QIAquick PCR purification kit (QIAGEN co., Japan). Then, these fragments were prepared for sequencing by BigDye Terminator v3.1 Cycle Sequencing kit (Applied Biosystems co., Japan) to obtain sequences with ABI310 sequencer. Additional primers, 457r (5’- CTCTTTGTTATTCCTTGAAATTTG -3’) and 500f (5’- TTTTTGGAAGCTCCATTGCGAG -3’), 1095r (5’- TGTTTACGAAATCTGGTTCTTTT -3’), and 1kbf (5’-ATGAGAAGAAACTCTCATGTCC-3’) were used to obtain inner sequences of the *rpl16* gene. Single insertions of 5 bp at nucleotides 7998/7999, or double insertions of 5 bp at nucleotides 7998/7999 and nucleotides 8197/8198, have been reported in flanking sequences of chloroplast encoding ORF, ORF100 (Takahashi et al. 2008). These insertions were detected only in *O. meridionalis*. The core collection and new accessions were primarily confirmed by sequencing. A pair of primers, ORF100-INDE were used to amplify the sequencing templates. Another pair of primers, ctDNA336 was used to amplify the region for comparison by electrophoresis as ORF100-INDEL (Figure 2). All ctDNA markers were amplified with NEB Taq using the buffer supplied by the manufacturer (NEB co., Japan). The mitochondrial DNA of *O. meridionalis* has aligned to the Nipponbare reference sequence (AB076665 and AB076666) by using whole genome data reported (Waters et al. 2012). A large unaligned region was presumed to be a deletion fragment. The deletion of the mtDNA INDEL was amplified. The amplification was carried out under the following conditions: 98°C for 3 min, 35 cycles of 98°C for 10 sec, 64°C for 30 sec, and then 72°C for 5 min, followed by 72°C for 5 min as an extension step with iProof Taq polymerase using the buffer supplied (Bio-Rad Co., USA).

### INDEL and SSR markers

Partial genomic sequence data for 21 *indica* cultivars, 18 tropical *japonica* cultivars, 32 accessions of *O. rufipogon*, one from *O. merdionalis* (IRGI101148), and one from *O. barthii* (IRGC104119) were used to develop nuclear DNA INDEL markers, with sequence information which had been kindly provided from published data (Molina et al. 2011). Five sequenced fragments were used to design nuclear DNA INDEL markers, as listed in Table 2, distinguishing fragments that originated from *O. meridionalis* as deletion types (Figure 4). INDEL10 was recognized as a DNA transposable element related to *Stowaway* (Bureau and Wessler 1994). These fragments shared 98% homology with each other. There was no insertion among the 18 accessions of *O. meridionalis* we used, including INDEL10 and others. In addition, all accessions of Asian *O. rufipogon* carried insertions.

Seven SSR loci were also designed based on sequence information from the above materials. All primer sequences are shown in Table 2. Ampliqon-Taq (Ampliqon Co., Denmark) was used for amplification with the manufacturer’s supplied buffer. PCR conditions were 94°C for 3 min, 30 rounds of 94°C for 10 sec, 55°C for 30 sec, and 72°C for 30 sec, followed by 72°C for 5 min for post-heating. Amplified products were genotyped using 6% denatured sequencing gels with a general silver staining method based on the SILVER SEQUENCE™ DNA Sequencing System (Promega Co., USA).

### Data analysis

The GenAlEx 6.2 software package (Peakall and Smouse 2006) was used for evaluating genetic variations in loci and populations, including the number of different alleles per locus (*Na*), *Fst*, observed heterozygosity (*Ho*), and expected heterozygosity (*He*). Dendrograms were constructed using the neighbor-joining method based on Nei’s unbiased genetic distances using the Populations 1.2.30 beta2 program (http://bioinformatics.org/populations/). Bootstrap values were assessed with 1000 replicates. All dendrograms were drawn by TreeExplorer (Tamura et al. 2011).

## Competing interests

The author declares that they have no competing interests.

## Author’s contributions

MA, YK, KT, KI, IN, TS, Y-IS, and RI organized and worked to collect new accessions in Australia and evaluate their life history. JF and MP aligned DNA sequences to set up INDEL markers specified species identification. DC and BS contributed to preparing, evaluating, and managing herbarium specimens. DW, RH, KO, RI provided NGS data to identify SNP and deletions. YH sequenced *rpl16* gene. SSR genotyping was conducted by YK. RI performed population genetics and INDEL genotyping. All authors contributed to the manuscript.

## Supplementary Material

Additional file 1: Figure S1 Polymorphism characterizing the chloroplast genome of Oceania wild rice. a. SNP found in the *rpl16* gene of the ctDNA genome of Asian *O. rufipogon*, Australia *O. rufipogon*, and *O. meridionalis*. T-A substitution (676 nt) and C insertion between 770 and 771nt in the 1^st^ intron, C-T substitutions at 1295 nt and 1406 nt. b. Phylogenetic tree created by the NJ method based on using *rpl16* sequences of *O. sativa* cv. Nipponbare, *O. rufipogon*, and *O. meridionalis*. The substitution rate is indicated as in the bar below. Click here for file

Additional file 2: Figure S2 Alignment of nuclear DNA INDEL markers. Panels a to e show sequences of INDEL5, 6, 7, 8, 9, and 10 with corresponding sequences in Jpn1, Jpn2, Jpn3, P27, and W1300. Except for INDEL10, they did not show any resemblance to transposable elements. INDEL10 was a *Stowaway* transposable element. Insertion types at the five INDEL loci, were only found among Asian species, *O. sativa* and *O. rufipogon*. Click here for file

Additional file 3: Figure S3 Jpn2 individuals inhabits through dry season. a. Jpn2 site in August, 2011, b.young shoot and roots emerging out from nodes. Click here for file
