# Supplementary figures and images for: Molecular relationships between Australian annual wild rice, Oryza meridionalis, and two related perennial forms
Source: Rice (N Y). 2013 Oct 28;6(1):26. doi: 10.1186/1939-8433-6-26 (PMC3874672; doi:10.1186/1939-8433-6-26)

## Slide 1
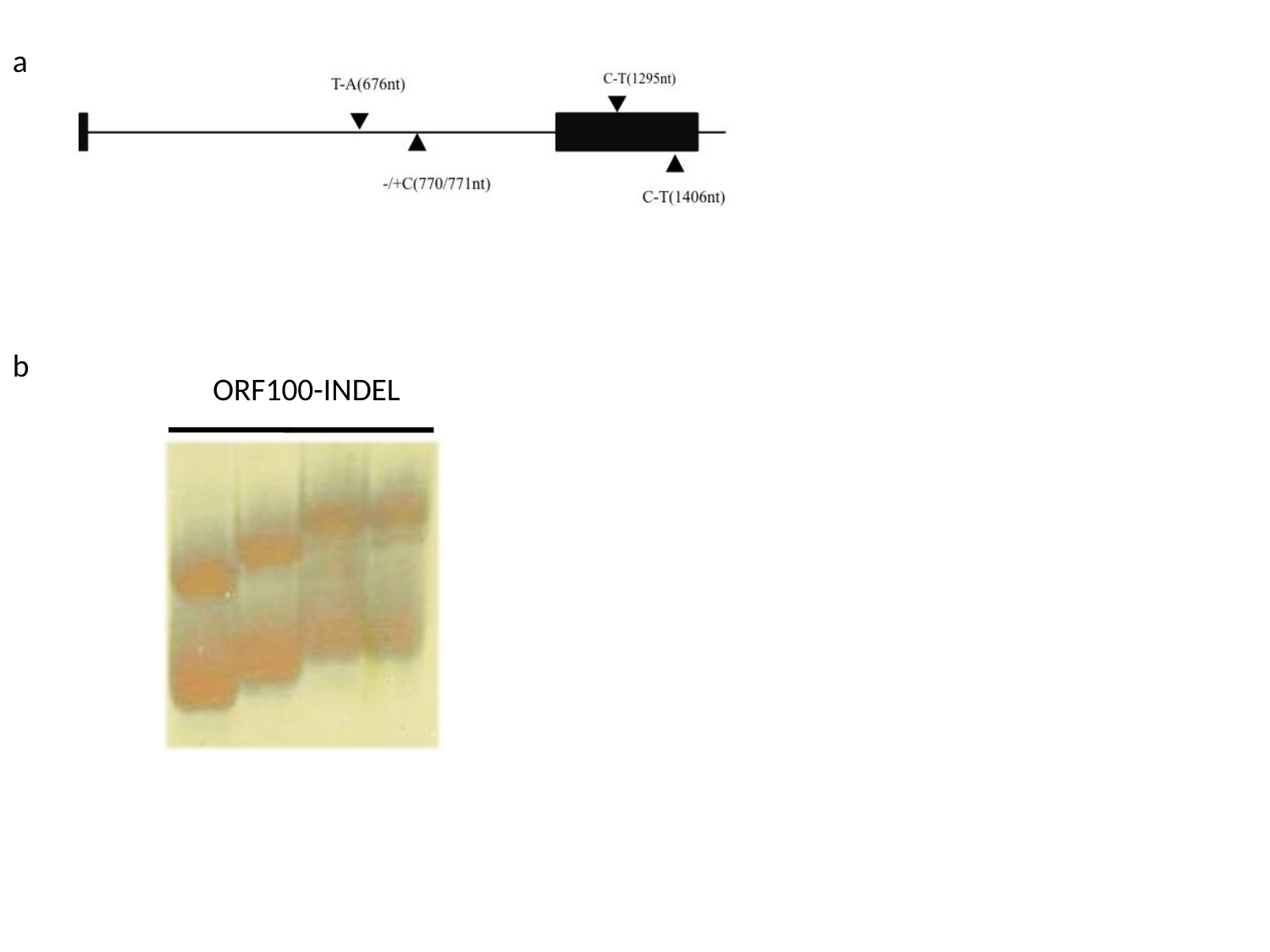

a
b
ORF100-INDEL

Supplement: Additional file 1: Figure S1 — Polymorphism characterizing the chloroplast genome of Oceania wild rice. a. SNP found in the rpl16 gene of the ctDNA genome of Asian O. rufipogon, Australia O. rufipogon, and O. meridionalis. T-A substitution (676 nt) and C insertion between 770 and 771nt in the 1st intron, C-T substitutions at 1295 nt and 1406 nt. b. Phylogenetic tree created by the NJ method based on using rpl16 sequences of O. sativa cv. Nipponbare, O. rufipogon, and O. meridionalis. The substitution rate is indicated as in the bar below. [file 1939-8433-6-26-S1.pptx]

## Slide 1
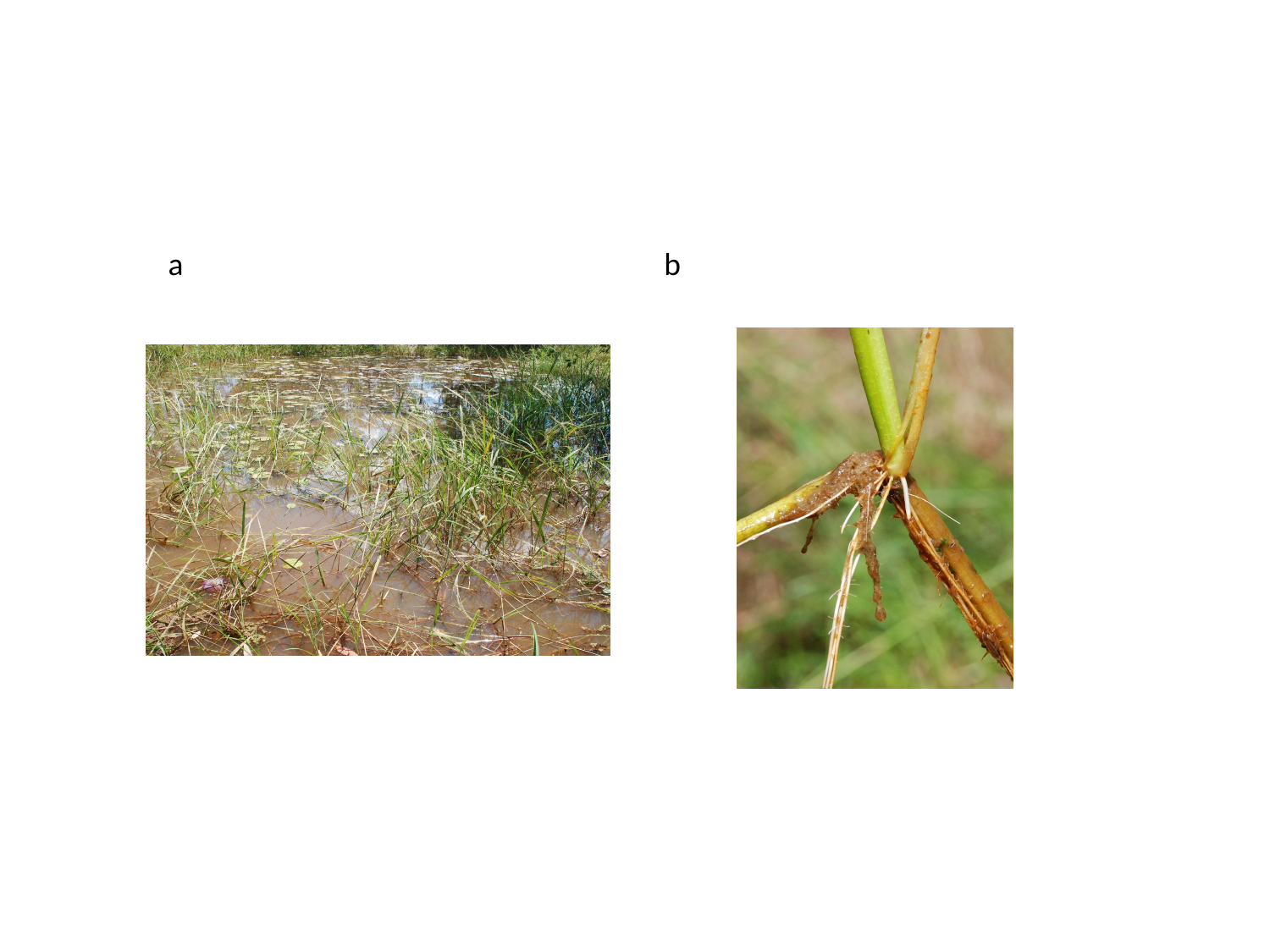

a b

Supplement: Additional file 3: Figure S3 — Jpn2 individuals inhabits through dry season. a. Jpn2 site in August, 2011, b.young shoot and roots emerging out from nodes. [file 1939-8433-6-26-S3.pptx]
