# Supplementary material for: Molecular relationships between Australian annual wild rice, Oryza meridionalis, and two related perennial forms
Source: Rice (N Y). 2013 Oct 28;6(1):26. doi: 10.1186/1939-8433-6-26 (PMC3874672; doi:10.1186/1939-8433-6-26)
Supplement: Additional file 2: Figure S2 — Alignment of nuclear DNA INDEL markers. Panels a to e show sequences of INDEL5, 6, 7, 8, 9, and 10 with corresponding sequences in Jpn1, Jpn2, Jpn3, P27, and W1300. Except for INDEL10, they did not show any resemblance to transposable elements. INDEL10 was a Stowaway transposable element. Insertion types at the five INDEL loci, were only found among Asian species, O. sativa and O. rufipogon. [file 1939-8433-6-26-S2.pptx]

## Slide 1
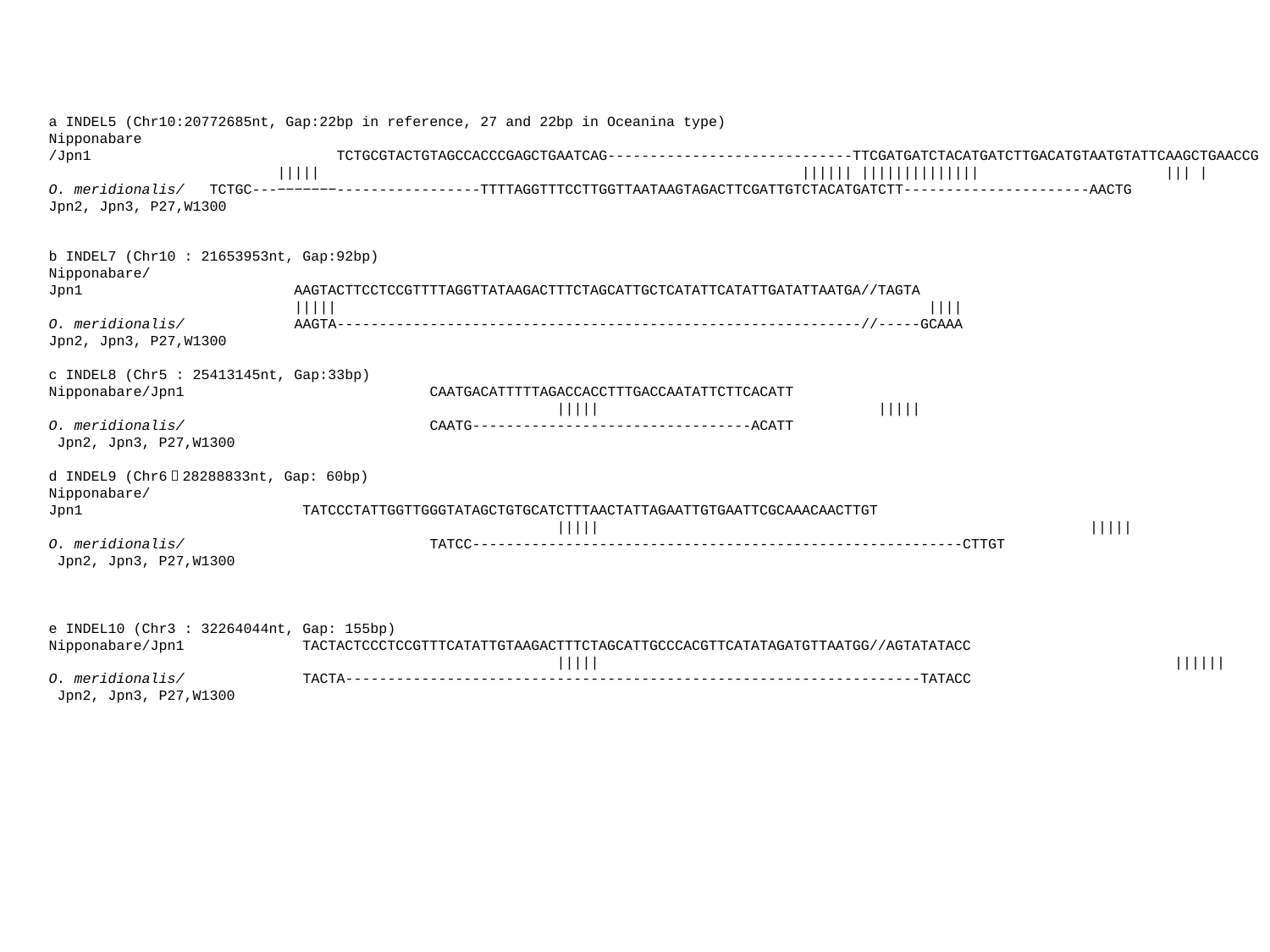

a INDEL5 (Chr10:20772685nt, Gap:22bp in reference, 27 and 22bp in Oceanina type)
Nipponabare
/Jpn1 		 TCTGCGTACTGTAGCCACCCGAGCTGAATCAG-----------------------------TTCGATGATCTACATGATCTTGACATGTAATGTATTCAAGCTGAACCG
	 |||||　　　　　　　　　　　　　　　　　　　　　　　　　　　　　　 　　　|||||| |||||||||||||| ||| |
O. meridionalis/ TCTGC---−−−−−−−-----------------TTTTAGGTTTCCTTGGTTAATAAGTAGACTTCGATTGTCTACATGATCTT----------------------AACTG
Jpn2, Jpn3, P27,W1300
b INDEL7 (Chr10 : 21653953nt, Gap:92bp)
Nipponabare/
Jpn1 AAGTACTTCCTCCGTTTTAGGTTATAAGACTTTCTAGCATTGCTCATATTCATATTGATATTAATGA//TAGTA
 ||||| ||||
O. meridionalis/ AAGTA--------------------------------------------------------------//-----GCAAA
Jpn2, Jpn3, P27,W1300
c INDEL8 (Chr5 : 25413145nt, Gap:33bp)
Nipponabare/Jpn1 		CAATGACATTTTTAGACCACCTTTGACCAATATTCTTCACATT
				||||| |||||
O. meridionalis/ 		CAATG---------------------------------ACATT
 Jpn2, Jpn3, P27,W1300
d INDEL9 (Chr6：28288833nt, Gap: 60bp)
Nipponabare/
Jpn1 TATCCCTATTGGTTGGGTATAGCTGTGCATCTTTAACTATTAGAATTGTGAATTCGCAAACAACTTGT
				||||| |||||
O. meridionalis/ 		TATCC----------------------------------------------------------CTTGT
 Jpn2, Jpn3, P27,W1300
e INDEL10 (Chr3 : 32264044nt, Gap: 155bp)
Nipponabare/Jpn1 TACTACTCCCTCCGTTTCATATTGTAAGACTTTCTAGCATTGCCCACGTTCATATAGATGTTAATGG//AGTATATACC
				||||| ||||||
O. meridionalis/ TACTA--------------------------------------------------------------------TATACC
 Jpn2, Jpn3, P27,W1300
